# Emergence and Control of African Swine Fever

**DOI:** 10.3390/pathogens14030283

**Published:** 2025-03-14

**Authors:** Lauro Velazquez-Salinas

**Affiliations:** National Bio and Agro-Defense Facility (NBAF), Agricultural Research Service, U.S. Department of Agriculture, Manhattan, KS 66502, USA; lauro.velazquez@usda.gov

African swine fever (ASF) is a highly lethal and contagious viral disease found in domestic pigs, wild boars, and wild suids, and it has significant economic consequences [1]. This disease is currently endemic in many parts of the world, but the lack of an effective commercial vaccine for prevention and control makes the threat of ASF spreading to ASF-free regions more prominent [1]. The identification of ASF in the Americas for the first time in almost 40 years demonstrates the urgent need to develop novel countermeasures to control this disease [2]. This editorial aims to announce the completion (31/01/2025) of the Special Issue “Emergence and Control of African Swine Fever” (https://www.mdpi.com/journal/pathogens/special_issues/emergence_control_african_swine_fever) (accessed on 13 March 2025). A total of 20 publications are included in this Special Issue. These publications can be classified into the categories of control (vaccination, drugs, disinfectants, and immunology) (*n* = 6), epidemiology (*n* = 6), pathogenesis (*n* = 5), and diagnostics (*n* = 3). Below, I highlight some of the main findings published in this Special Issue. 

Currently, the use of live attenuated vaccines (LAVs) based on the deletion of specific viral genes appears to be one of the most promising strategies for controlling ASF [3]. However, multiple challenges are associated with the development of LAVs, such as the lack of adapted cell lines that support the replication of ASFV and the limited knowledge about the safety profile of LAV candidates in the long term. In light of this, Ramirez-Medina et al. (2024) reported the ability of IPKM (Immortalized Porcine Kidney Macrophage) cells to be used in the production of stocks for LAVs, which only replicate in primary swine macrophages. The authors showed the efficacy of these cells in producing the LAV candidate ASFV-G-Δ9GL/ΔUKp10, which was able to protect pigs via the virulent ASFV-G (parental strain). On the other hand, Borca et al. (2023) documented the absence of the long-term (180 days) residual virulence of ASFV-G-∆I177L, highlighting the safety profile of this LAV candidate.

In terms of the protective mechanisms induced by the LAV candidates of ASFV, Silva et al. (2022) contributed a study using ASFV-G-Δ9GL/ΔUK and ASFV-G-ΔI177L, showing the relevance of neutralizing antibodies during protection induced by these LAV candidates. Also, Wu et al. (2024) published an interesting review on the implications of T/B-cell epitopes and vaccine adjuvants in the immune response and vaccine development of ASFV.

As an alternative mechanism of control of ASF, Jackman et al. (2023) explored the significance of glycerol monolaurate (GML) in inhibiting the infection of a highly virulent strain of ASFV (isolate Armenia/07) in porcine macrophages, showing a > 99% decrease in viral infectivity. Furthermore, Frost et al. (2023) evaluated the efficacy of 24 commercial disinfectants, demonstrating the importance of disinfectants containing formic acid, phenolic compounds, and oxidizing agents in decreasing the viral titers of ASFV.

The infectious cycle of ASFV in nature involves complex epidemiological dynamics that complicate the control and eradication of ASF [4]. Different epidemiologic studies were published in this Special Issue, giving more insights into the factors predisposing the circulation of ASFV worldwide. Schambow et al. (2023) conducted an epidemiologic assessment in the Dominican Republic, showing the correlation of low biosecurity practices with the presentation of outbreaks in backyard farms. Spatiotemporal analysis suggested the existence of an endemic pattern of ASF located in the central region of this country. In the Philippines, Hsu et al. (2023) carried out an epidemiological study to identify the factors influencing the diagnosis, spread, and control of ASF in this country. This study highlights the relevance of timely reporting and enhanced biosecurity measures to manage ASF outbreaks in the Philippines. Additionally, Moskalenko et al. (2024) evaluated the awareness and perceptions of pig keepers about ASF in Ukraine, demonstrating the necessity of improving communication strategies about ASF in this country.

Two interesting studies involving molecular epidemiology were published in this special edition. On the one hand, Okwasiimire et al. (2023) conducted a study in Uganda, confirming the circulation of the ASFV genotype IX in domestic pigs in this country. Whole genome sequencing analysis revealed details about the evolution of this genotype, indicating the necessity of characterizing current viral strains circulating in Uganda to identify potentially new clinical phenotypes. On the other hand, Mazloum et al. (2022) conducted the genetic characterization of the ASFV isolates associated with genotype II in the Russian Federation between 2013 and 2017. Using the central variable region (CVR) of the ASFV gene B602L, it was shown that viral strains that circulate in twenty-three different regions within the Russian Federation have diverged into six different phylogenetic groups. The authors propose using the CVR of B602L to conduct future epidemiology studies in Europe and Asia.

Finally, in this category of epidemiology, Richter et al. (2023) presented an epidemiologic analysis comparing the circulation patterns of ASF in wild boars in Germany (Saxony) and Latvia. The results of this comparison indicated that the rapid implementation of new control strategies based on previous experiences from other countries might have improved Saxony’s response to ASF in wild boars.

Understanding the pathogenesis of ASFV is a key aspect in controlling the disease. Indeed, identifying reliable experimental models that replicate the clinical outcomes observed in the field is an important aspect to be determined by pathogenesis studies [5]. In this sense, Olesen et al. (2025) contributed an interesting study to this Special Issue, in which they evaluated the dose of the ASFV strain (ASFV POL/2015/Podlaskie strain) required to establish infections in pigs following oral uptake (natural route of infection). The authors concluded that compared with the intranasal route, higher doses are needed to establish an infection via the oral route. These results highlight the implications of control strategies for ASF using baited vaccines containing LAVs. In another study using the oral route of inoculation, Nguyen et al. (2023) conducted a pathogenies study in pigs to characterize the ASFV strain (VNUA/HY/ASF1) associated with the first reported outbreak of ASF in Vietnam. Pigs infected with VNUA/HY/ASF1 produced disparate clinical outcomes (acute and subacute), producing clinical signs between 4 and 14 days post infection (dpi) and causing the death of the pigs between 10 and 27 dpi. This study provides relevant information about the pathogenesis of ASFV using a natural exposure model.

Some years after the emergence of the highly virulent ASFV genotype II in 2007, clinical outcomes observed in the field started varying from fatal acute to subclinical, indicating the existence of low-virulence variants [6]. In this sense, Avagyan et al. (2024) conducted a study to characterize ASFV variants producing chronic and persistent infections in pigs, concluding that chronic forms of ASF are associated with a decreased immune response and lower infectious titers found in the blood and tissues of the infected pigs.

Similarly, Sehl-Ewert et al. (2022) conducted a study characterizing the viral variants emerging from the ASFV lineage genotype II circulating in wild boars in Germany. The pathological analysis of carcasses obtained from naturally infected wild boars showed potential disparate levels of virulence among natural ASFV variants circulating in Germany. This study highlights the relevance of variants producing extended disease outcomes and their potential impact on shedding and transmission.

Furthermore, in this category of pathogenesis, Li et al. (2022) published a transcriptomic analysis of macrophages infected with ASFV. The results of this study emphasized the potential role of the NF-κB signaling pathway in the early stage of infection with ASFV in macrophages, providing more insights into the pathogenesis of ASFV.

Undeniably, diagnostics play a key role in the control of ASF [7]. In this sense, research associated with the development of new diagnostic methodologies that improve the detection of ASFV constitutes an important milestone in the rapid and accurate detection of this disease. As part of this Special Issue, Friedrichs et al. (2024) evaluated qPCR protocols for ASFV diagnosis in wild boars using semen as a target sample. As a result, the authors published a workflow that detected the genome of ASFV in the semen of wild boars infected as early as two days post infection. Another qPCR protocol was published by Shi et al. (2023). These authors developed a triplex crystal digital PCR able to detect and rule out the presence of the ASFV genes B646L, MGF505-2R, and I177L. Notably, the last two genes are implicated in the development of LAVs. This condition highlights this design as a diagnostic tool capable of differentiating vaccinated from infected animals with ASFV.

Lastly, Watanabe et al. (2023) developed an indirect enzyme-linked immunosorbent assay (ELISA) using recombinant p11.5 protein to detect antibodies against ASFV. By using a collection of serum samples from pigs and wild boars experimentally infected with multiple ASFV strains, the authors demonstrated the value of this serological method in detecting antibodies early during infection.

In conclusion, the studies published in this Special Issue on the emergence and control of African swine fever contribute to improving the knowledge of some critical aspects of this complex disease. I encourage the scientific community of ASFV to review these published studies.

## Data Availability

No primary data are included in this article.
